# Defining ‘science-based targets’

**DOI:** 10.1093/nsr/nwaa186

**Published:** 2020-08-25

**Authors:** Inger Andersen, Naoko Ishii, Thomas Brooks, Cynthia Cummis, Gustavo Fonseca, Astrid Hillers, Nicholas Macfarlane, Nebojsa Nakicenovic, Kevin Moss, Johan Rockström, Andrew Steer, Dominic Waughray, Caroline Zimm

**Affiliations:** United Nations Environment Programme, Kenya; Global Environment Facility, USA; International Union for Conservation of Nature, Switzerland; World Resources Institute, USA; Global Environment Facility, USA; Global Environment Facility, USA; International Union for Conservation of Nature, Switzerland; International Institute for Applied Systems Analysis, Austria; World Resources Institute, USA; Potsdam Institute for Climate Impact Research, Germany; Conservation International, USA; Stockholm Resilience Centre, Sweden; World Resources Institute, USA; World Economic Forum Centre for Global Public Goods, Switzerland; International Institute for Applied Systems Analysis, Austria

The 2015 Paris Agreement to keep global warming well below 2°C above pre-industrial levels and aim towards limiting warming to 1.5°C marked a watershed in planetary governance, for two reasons. First, of course, it set an explicit, quantitative target for sustainability with strong support from science, in a clearer way than had ever been done before. Second, perhaps even more important, this target is structured in a way that it can be disaggregated across the sectors of society which will need to take action to achieve it. This includes not only the nations who agreed on the target in the first place, but also non-state actors, such as cities, regional governments and the private sector. We see the prospect for each component of society to ‘do their bit’ towards ameliorating climate change as a fundamentally important precedent for global governance. With the upcoming 2020 timelines for a number of the targets under the 2030 Agenda for Sustainable Development, as well as for the plans of a number of multilateral agreements, the world has a grand opportunity to replicate this concept of ‘science-based targets’.

**Table 1.** Examples of global environmental goals as overall science-based targets.

	Achievability	Quantification	Rationale for level
Paris Agreement 2–1.5°C target	Yes	Yes	Yes
Strategic Plan for Biodiversity 2011–2020	Yes	Yes	Yes
Land Degradation Neutrality Target	Yes	Yes	Yes
SDG 14	Yes	No	No
SDG 15	Yes	Partial	Partial

The first goal of the Paris Agreement, the mission of the Strategic Plan for Biodiversity 2011–2020, and the Land Degradation Neutrality Target exhibit all three of the characteristics defined here for being considered ‘overall science-based targets’—achievability, quantification and rationale. Sustainable Development Goals 14 and 15 have the first of these, and two of the clauses of the latter also have explicit quantification and underlying rationale.

The term ‘science-based targets’ has burst into the discourse of the science– policy interface for sustainability over recent months. Rockström *et al.* [1] used the term to describe the targets under the Paris Agreement. An entire Science-based Targets Initiative has been established by the Carbon Disclosure Project, United Nations Global Compact, World Resources Institute and World Wildlife Fund to guide companies in setting science-based emissions reductions targets for climate change. The initiative has reached critical mass, illustrative of the rapid growth in application of the term by many non-governmental organizations and governments. As another example, the charity Oxfam emphasizes ‘setting and implementing science-based targets’ in their corporate engagement, while in 2016 the International Union for Conservation of Nature's Resolution 96 highlighted the term in the context of biodiversity conservation.

However, it has become apparent that the term is being used in widely different ways, which is generating substantial confusion. Here, we therefore seek to define what ‘science-based’ means in relation to ‘science-based targets’, and to differentiate between overall science-based targets (for the world) and specific science-based targets (for individual entities). We do not seek to explore the experiences, challenges and impacts of the establishment of science-based targets in practice; such work is underway through a wide range of processes, and will be reported on in due course.

## WHAT DOES IT MEAN TO BE ‘SCIENCE-BASED’?

Setting targets for addressing major planetary concerns is an essential prerequisite for concerted global action (both inside and outside multilateral environmental agreements) and is necessarily a societal and political process, requiring negotiation and convergence among often-conflicting interests [2]. There is no such thing as a ‘scientific target’ applied in policy or business—operational targets are socio-political choices.

However, this is not to say that targets cannot be ‘science-based’. What, then, does it mean for a target for addressing major planetary concerns to be ‘science-based’? First, recall that ‘science’ is ‘the organised, systematic enterprise that gathers knowledge about the world and condenses the knowledge into testable laws and principles’ [3]. Building from this, we propose the following characteristics as defining ‘science-based targets’:

- Analytical evidence suggests that the achievement of the target is a biophysical possibility within its specified time frame. This clearly does not mean that its achievement is a foregone conclusion; addressing cultural, political, social and economic constraints to achieving targets can be hugely challenging.
  But for a target to be science-based, it must be theoretically achievable.
- It must be possible to demonstrate—and test—the degree to which a target has been achieved. The target should be quantified, such that progress towards it is measurable. Such quantification could be in the form of an absolute value (e.g. ‘2°C above pre-industrial levels’) or a relative one (e.g. ‘halt the loss’, or ‘reduction by x%’).
- The target should be supported by a clear, analytical rationale for why it is set at this particular level. This might often be expressed in the form of a probability of achieving an ethical imperative (such as ending hunger or preventing extinction), or of reducing the risk of a negative outcome, such as transgression of a ‘planetary boundary’, to an acceptable level.

The first and second of these characteristics overlap with the characteristics of ‘SMART’ targets, i.e. targets that are specific, measurable, assignable, realistic and time-related [4]. However, while science-based targets are necessarily ‘SMART’, the converse is not necessarily the case, because SMART targets are not necessarily underpinned by a scientific rationale.

## OVERALL SCIENCE-BASED TARGETS

Overall science-based targets are those established through intergovernmental process at the level of the entire planet. The best-known example comes from the Paris Agreement under the United Nations Framework Convention on Climate Change, which sets an overall science-based target of ‘keeping a global temperature rise this century well below 2 degrees Celsius above pre-industrial levels and to pursue efforts to limit the temperature increase even further to 1.5 degrees Celsius’. While the 2°C target is of course a product of political negotiation, and is based on decades of climate science and requires accepting what many consider to be unacceptable risks of negative consequences [5], it meets all three of the characteristics proposed above for being considered ‘science-based’ (Table 1) as well as all five characteristics of a ‘SMART’ target.

**Figure 1.**
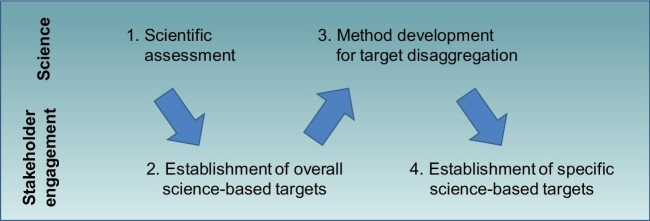
Schematic process for development of science-based targets. The establishment of overall science-based targets is informed by independent assessment and synthesis of the state of the science of a given planetary concern (1) and then negotiated through policy dialogue to reach global agreement (2). Once overall science-based targets have been established, scientific research into method development is then necessary to allow disaggregation of these across (3), allowing engagement across all sectors of society to set specific science-based targets and ensure implementation of actions to reach them (4).

A number of other overall science-based targets for addressing major planetary concerns have been set through intergovernmental processes. For example, the Strategic Plan for Biodiversity 2011–2020 sets a target to ‘take effective and urgent action to halt the loss of biodiversity in order to ensure that by 2020 ecosystems are resilient and continue to provide essential services’, while the United Nations Convention to Combat Desertification maintains a ‘Land Degradation Neutrality Target-Setting Programme’. Both of these share the characteristics outlined above (Table 1).

The highest level intergovernmental targets for addressing major planetary concerns are the Sustainable Development Goals (SDGs). These 17 goals encompass 169 targets; the degree to which both the goals themselves and their constituent targets are science-based is variable. For example, Goal 15 (‘Sustainably manage forests, combat desertification, halt and reverse land degradation, halt biodiversity loss’) is science-based (at least in its third and fourth components, where the verb ‘halt’ provides explicit quantification) while Goal 14 (‘Conserve and sustainably use the oceans, seas and marine resources’) is not science-based (Table 1).

## SPECIFIC SCIENCE-BASED TARGETS

Once overall science-based targets have been established, it becomes possible (and highly desirable) to also set specific science-based targets. These are derived through disaggregation of overall science-based targets, based on the extent of contribution of a given entity or sector towards causing a major planetary concern, such as greenhouse gas emissions by a given company [6]. They therefore identify the specific contributions that a given entity would need to achieve, such that if all such entities achieved equivalent targets, the overall science-based target would in turn be achieved. In other words, they establish the equitable division of responsibility of individual entities to meet an overall global target.

Accordingly, for the example of climate change, the draft ‘Science-based Target Setting Manual’ of the Science-based Targets Initiative defines science-based targets as those ‘in line with the level of decarbonization required to keep global temperature increase below 2°C compared to pre-industrial temperatures’. A recent scoping by the World Resources Institute and Mars Incorporated, *From Doing Better to Doing Enough: Anchoring Corporate Sustainability Targets in Science*, discusses the potential for extension of specific science-based target approaches from climate change to freshwater, concluding that it would be challenging—but possible.

The entities in question could be any kind of societal unit. The Science-based Targets Initiative focuses on businesses as the relevant unit, and where possible, determines the target for the individual business via sector targets first. Such applications in the private sector cannot substitute for public policy [7], but they could both complement and stimulate it. Thus, countries could also comprise the units, such that specific science-based targets could guide appropriate national targets to be set in Nationally Determined Contributions under the Paris Agreement, or in National Biodiversity Strategies and Action Plans under the Convention on Biological Diversity. Likewise, regions and sub-regions comprising similarly-situated countries could be considered as units to develop specific science-based targets applicable to the common successes, challenges and opportunities among the member states (an example could be the ASEAN Socio-Cultural Blueprint 2025). The same approach could be applied to the level of sub-national units, e.g. states or provinces, cities or municipalities. Moreover, the investor community will likely increasingly draw from specific science-based targets to inform their allocation of funds, in the same way that they have benefitted from corporate greenhouse gas emissions disclosure efforts and related company commitments to reduce emissions.

While more than 900 companies have adopted science-based targets for mitigating climate change, Bjørn *et al.* [8] have shown more broadly that the uptake of such approaches has been relatively limited to date. This may be because methods for disaggregation are in their infancy, given the challenge of linking drivers to responsibility around our interconnected planet. However, advances in life cycle assessment [9,10], environmentally-extended input-output analysis [11,12], and similar techniques are opening great potential in allowing such specific science-based targets to be set for a range of planetary concerns, across multiple levels of society.

## THE PROCESS FOR ADVANCING SCIENCE-BASED TARGETS

What are the mechanisms for development of such overall and specific science-based targets? We envision four transdisciplinary activities, two each focused on either side of the interface between science and engagement with stakeholders (see Fig. 1). The first step must be a robust, independent assessment and synthesis of the state of the science; the assessment reports of the Intergovernmental Panel on Climate Change are the best-known example. Second, on the policy side, comes dialogue among countries (and stakeholders) to agree on overall science-based targets. Debate in the Conference of the Parties of the United National Framework Convention on Climate Change, yielding the Paris Agreement, exemplifies this. Dialogues across organized regional and subregional groups also helps to facilitate consensus at the global level, by consolidating the broad range of country-by-country perspectives. With overall science-based targets established, scientific inquiry and research into practicability of methods is then necessary to develop the methods for disaggregation of these into specific science-based targets, drawing from relevant expertise across global, regional and local scales. The research agenda around measurement of carbon footprints provides an example from the climate change perspective. Finally, engagement across all sectors of society—communities, cities, companies, as well as countries—is essential to set such specific science-based targets and ensure implementation of action towards achieving them. The Science-based Targets Initiative is an example of a platform for such dialogue. Most recently, the Science-based Targets Network has assembled to support the private sector and cities in establishing specific science-based targets for multiple dimensions of environmental sustainability.
